# Assessment of Unmanned Aerial Vehicles Imagery for Quantitative Monitoring of Wheat Crop in Small Plots

**DOI:** 10.3390/s8053557

**Published:** 2008-05-26

**Authors:** Camille C. D. Lelong, Philippe Burger, Guillaume Jubelin, Bruno Roux, Sylvain Labbé, Frédéric Baret

**Affiliations:** 1 CIRAD, UMR TETIS, 500 Rue J.-F. Breton, 34093 Montpellier Cedex 5, France; 2 INRA, UMR 1248 AGIR, Chemin de Borde Rouge, BP52627, 31326 Castanet Tolosan Cedex, France; 3 Nev@ntropic, 16 bis Avenue du Quatorze Juillet, 97300 Cayenne, France; 4 L'Avion Jaune, Minéa Incubation, 361 Rue J.-F. Breton, BP5095, 34196 Montpellier Cedex 5, France; 5 Cemagref, UMR TETIS, 500 Rue J.-F. Breton, 34093 Montpellier Cedex 5, France; 6 INRA, UMR 1114 EMMAH, Domaine St Paul, Site Agroparc, 84914 Avignon Cedex 9, France

**Keywords:** Imagery, Multispectral, Precision Farming, UAV

## Abstract

This paper outlines how light Unmanned Aerial Vehicles (UAV) can be used in remote sensing for precision farming. It focuses on the combination of simple digital photographic cameras with spectral filters, designed to provide multispectral images in the visible and near-infrared domains. In 2005, these instruments were fitted to powered glider and parachute, and flown at six dates staggered over the crop season. We monitored ten varieties of wheat, grown in trial micro-plots in the South-West of France. For each date, we acquired multiple views in four spectral bands corresponding to blue, green, red, and near-infrared. We then performed accurate corrections of image vignetting, geometric distortions, and radiometric bidirectional effects. Afterwards, we derived for each experimental micro-plot several vegetation indexes relevant for vegetation analyses. Finally, we sought relationships between these indexes and field-measured biophysical parameters, both generic and date-specific. Therefore, we established a robust and stable generic relationship between, in one hand, leaf area index and NDVI and, in the other hand, nitrogen uptake and GNDVI. Due to a high amount of noise in the data, it was not possible to obtain a more accurate model for each date independently. A validation protocol showed that we could expect a precision level of 15% in the biophysical parameters estimation while using these relationships.

## 1. Introduction

The American National Research Council [1] defined precision agriculture (or precision farming) as “a management strategy that uses information technology to bring data from multiple sources to bear on decisions associated with crop production”. It is commonly admitted that it encompasses all the techniques and methods of crop and field information gathering that help taking into account in crop management the local and site-specific heterogeneity [2-3-4-5-6]. Remote sensing image products, such as biophysical parameters maps for instance, have proven to be of high information content for that purpose, especially thanks to their spatial dimension [7-8-9]. Vegetation indexes, derived from accurately calibrated remote sensing images, can help producing such maps by means of empirical or modelled relationships [10-11-12-13-14-15]. They are now widely used by the remote sensing community especially to provide coupled agronomical and spatial information about cereal crop status like wheat [e.g. 16-17-18-19-20-21-22-23]. Such products are then often assimilated in crop models to derive more complex crop stress information [24] or even directly integrated into a Geographical Information System for precision practices management [25-26].

Because it has rapidly appeared that satellite sensors did not meet the requirements in increasing image temporal frequency and spatial resolution for such application like crop monitoring, many airborne photographic or video systems have been developed to compensate spatial lack of opportunities [27 - 28 - 29 - 30]. On the other hand, in-field devices (like onboard traction-engine) are also relevant for site-dedicated systems at affordable costs [e.g. 31-7]. However, they are difficult to move from one site to another, have a small mapping swath capability, and are often only available during cropping activities. Cameras mounted on ultra light aircrafts or even unmanned aerial vehicles (UAVs) are now a good compromise between the high performances that such sensors can provide and cost-effectiveness of data acquisition. First developed for defence applications, especially as a target or weapon reconnaissance platform [e.g. 32-33-34], these little vectors are now catching the attention of a wider range of image-users like archaeologists [e.g. 35] or forest-fires detectors [e.g. 36]. The increasing capabilities of such remotely controlled vehicles, of on-board attitude-control or positioning systems, and of digital cameras technology, combined to the improvements of remote sensing techniques, have recently pushed the UAV into the precision farming field. For instance, Herwitz et al. [37] have shown that multispectral sensors on board UAVs can provide spectral indexes to be related with the mature yield of coffee crops. Sugiura et al. [38] even developed a complex platform dedicated to corn agricultural management, flying on board an unmanned helicopter, with a whole image processing and LAI maps production adapted chain. Even though their system is very efficient, onboard technology is quite expensive. It includes not only the imaging sensor itself (composed of three distinct matrices), but also inertial and geomagnetic sensors, GPS receivers, and data acquisition computer. Fortunately, many laboratories also seek simpler but efficient systems that could meet vegetation monitoring requirements with less constraints [e.g. 39]. In this context, we propose in this paper to analyse the potential for precision farming of a cost-effective system composed of simple digital cameras modified for multispectral acquisitions, fitted on board small UAVs.

We will focus on the case study of wheat, cropped in small plots, over the growing season. We will first describe the agronomical trials set for this purpose, and the ground truth data acquired as the reference information on wheat crops. Then we will explain the principle of our image acquisition device. After that, the data pre-processing protocol will be exposed, followed by the method of biophysical parameters estimation. Finally, we will discuss the validity of the derived relationships and the precision of the produced maps, to conclude about the relevance of such remote sensing platform for wheat crop monitoring.

## 2. Material

### 2.1 Trial plots

**Figure 1.**
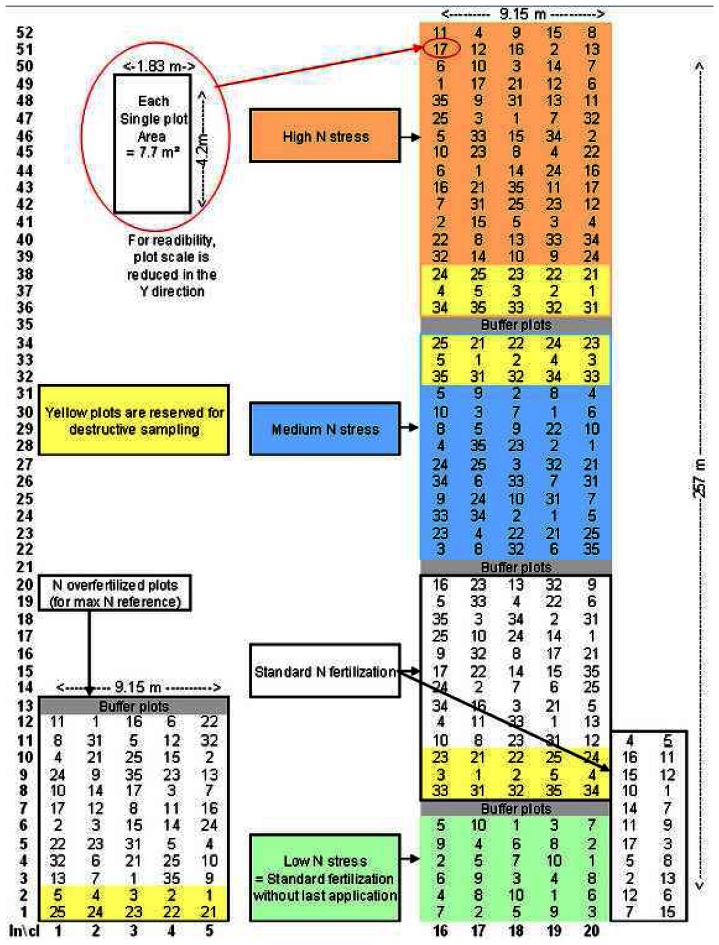
Sketch-plot of the wheat trial studied in this paper. Numbers indicate the sown genotype in the micro plot.

A rainfed variety trial of wheat was conducted in the 2004/2005 growing season at the French National Agronomical Research Institute (INRA) station in Auzeville, near Toulouse (South-West of France). This trial included 332 micro-plots (cf. Figure 1), each one covering 7.7 square meters (4.2 m *1.83 m, 16 cm apart).

Seventeen genotypes were sowed into these micro-plots: fifteen durum-wheat (Triticum Turgidum L. var. durum) and two bread-wheat (T. Aestivum L.), with three seeding densities: 170, 250 (the standard practice) and 390 seeds per square metre. The statistical design was a set of five sub trials established in the same field, containing a randomised design of three or four replicates of each specific genotype at different seeding densities.

Five different levels of Nitrogen availability were applied: 1- a standard N fertilization, 2- a standard N fertilization without the last application in the season (low N stress), 3- a medium level of N stress, 4- a high level of N stress, 5- an over fertilization. Not all combinations of genotype/N level/seeding density were tested.

For four N-levels, additional plots were included for destructive sampling during the growing season, for five genotypes (four durum wheat and one bread wheat) with the three densities.

This trial allows displaying a wide range of biomass, leaf area, and nitrogen uptake over the field and the growing season, useful to test remote sensing relevance for wheat monitoring.

### 2.2. Ground-truth data

Biophysical parameters were measured on the additional plots at dates close to each UAV flyby: 30 plots on the 23^rd^ of March, 46 plots on the 6^th^ of April, and 61 plots on the 27^th^ of April and the 18^th^ of May, 2005. Heading occurred around the 10^th^ of May and flowering around the 18^th^ of May. On each sampled plot, the number of plants at early tillering and the number of spikes per square meter were counted on four rows of one meter. To avoid premature destruction of the sample plots and to represent correctly the whole plot, plants were picked at four different places in the plot at each date. Leaf area index (LAI, in square metre per square metre) was assessed on a sub-sample with a Licor planimeter. Fresh plants were oven-dried at 80°C to a constant weight and then ground to 0.5 mm. Total nitrogen content (NC) was determined using a LECO CHN-2000 analyzer (Dumas method). Shoot-biomass per square metre was estimated by multiplying stem number (or spike number after heading) per square metre by the mean stem weight. The total nitrogen uptake (QN, in kg N/ha) is then calculated by multiplying above ground biomass by total N content. An overall precision of 15% was estimated for these destructive measurements.

LAI and QN measured in the sampled micro plots over the season have a correlation coefficient less than 0.6: they are thus complementary to characterize wheat crops agronomical status.

In addition to quantitative measurements, descriptive data were recorded during the experiment, like the plant habit, height, vigour, and earring date.

### 2.3. UAV-airborne images

**Figure 2.**
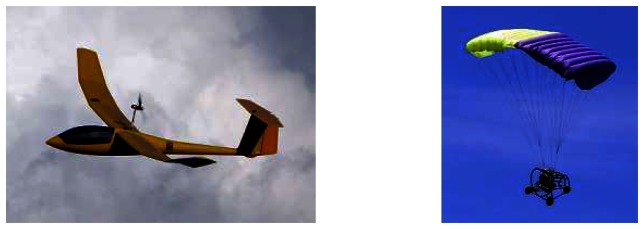
L'Avion Jaune's powered glider (left) and Pixy motorized parachute (right).

Imaging data were collected with sensors derived from standard cameras, fitted on two kinds of radio-controlled UAVs: a powered glider developed by the French corporation L'Avion Jaune (http://www.lavionjaune.fr), and a small “Pixy” motorized parachute developed by ABS-Aerolight and the French Research Institute for Development (IRD) (Figure 2). These different vehicles were used to test their respective agility and flight constraints, which we will not discuss here because it has no direct incidence on image production during this campaign.

The sensor was composed of a pair of similar digital cameras, available on the public market, but with some instrumental adaptations. Depending on the acquisition date, we used two different models:

- CANON EOS 350D: a reflex camera with 8 GigaPixels classical Bayer CCD-matrix splitting the light in three channels (Red, Green, and Blue).
- SONY DSC-F828: a reflex camera with 8 GigaPixels CCD-matrix splitting the light in four channels (Red, Green, Blue, and Cyan).

**Figure 3.**
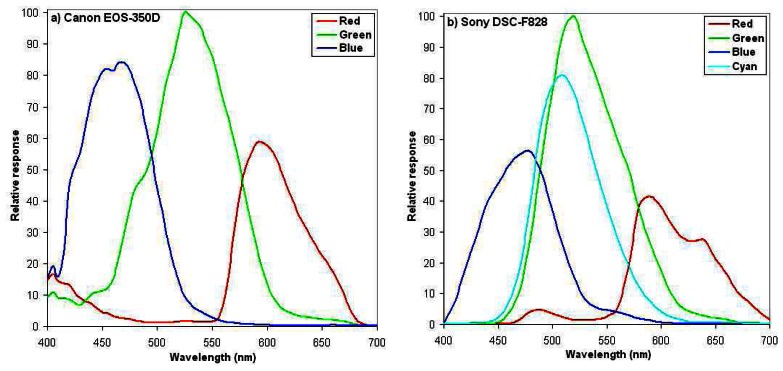
Spectral relative response of: a) the three Canon EOS-350D, and b) the four Sony DSC-F828 channels.

The information about the CCD photodiodes spectral sensibility is not available from the manufacturer, neither the Bayer matrix to be able to estimate the spectral correspondence of each camera produced channel [40]. We were thus obliged to perform afterwards an optical characterization in the ONERA laboratories in Toulouse to get the resulting relative spectral response of the camera. Figure 3 shows the spectral sensitivity of the Canon EOS-350D and the Sony DSC-F828, respectively. Bandwidths are then derived at the half-height of the response curve, and given at Table 1. Considering common satellite sensors wavelength coverage (see Table 1), these bands are quite close to those generally used in remote sensing, although slightly (10 to 30 nm) shifted to smaller wavelengths. They are thus correctly located to characterize the vegetation, even if the red band fits the negative slope of the chlorophyll absorption band rather than its minimum.

Each camera contains, in front of the CCD-matrix, a band-pass filter dedicated to catch only the visible part of the incident radiation and to stop the UV and infrared ones. But the CCD itself is sensible to infrared radiations up to 900nm. On one camera of each pair, we replaced this band-pass filter by an equivalent transparent glass slice to keep the same optical path than inside the other camera, and mounted on the objective a 715 nm high-pass filter (MaxMax trademark, model: X-Nite 715 Infrared) to block the visible part of the radiation. Finally, the red channels of these two modified cameras both provide an infrared sensor in the range 720-850 nm. This spectral range is also shifted to shorter wavelengths compared to common satellite sensors (see Table 1), but includes the relevant wavelengths to characterize the infrared plateau displayed by vegetation reflectance, just after the red-edge.

As the two cameras were mounted on the same axis and synchronized with a single trigger, the pair of standard plus modified cameras acquires the same image frame simultaneously. It thus composes a multispectral imager providing four spectral bands: blue, green, red, and near infrared, that are consistent with the more common remote sensing sensors bands. Considering that low-altitude flights performed by UAVs avoid strong interactions of measured reflected light with atmosphere, it provides scenes of higher radiometric homogeneity than aircraft or satellite sensors, and thus a better overall quality of signal. Nevertheless, other problems (e.g. geometrical) occur from the UAV use, especially because of the lack of motion and attitude control (that could provide an Inertial Unit for instance), and homemade sensors specifications that we will discuss in the next section.

The focal distance was fixed to a value between 7.9 and 8.0 mm. The shutter speed depended on the light conditions on acquisition day, set in general at 1/1000 second or 1/2000. The rate of image recording in this configuration was only 3 seconds for a “jpg” file, compared to 15 seconds for a “tiff” file. Thus, “tiff” storage was too long to capture the whole area of interest in a single flyby. However, the jpg format has the data compression inconvenient, which may add radiometric degradation, and which model for the used cameras was not available from the manufacturer. Therefore, we tested both jpg and tiff files recorded on the same views on the 14/04/2005: difference between these pairs showed that the mean radiometry is not altered inside a micro plot but can be strongly affected at the micro plot borders due to high frequency JPEG filtering method. We then decided to store the files in the “jpg” format and use a buffer when extracting the mean radiometry of a micro plot (see section 3.2).

We performed the six flights on the following dates: 28/02, 01/04, 14/04, 29/04, 25/05 and 08/06/2005. Solar zenith-angle was constrained between 40° and 50°, thanks to an acquisition time shifting from 1 to 3 pm (solar time) from date to date. As several photo frames might be necessary to cover the whole scene swath, all the acquisitions were done in a short single flyby to avoid light variations. At the end, a dozen of images were acquired at each date. Among them, we selected the smallest set of images among the less fuzzy or bloomed that allows covering the whole site: the less the number of images, the fewer inter-frame calibration bias while producing a mosaic. Finally, three (14/04, 08/06), four (28/02, 01/04, 25/05), or five (29/04) images were used per date to complete the scene.

## 3. Method

### 3.1. Pre-processing

#### 3.1.1. Vignetting correction

Vignetting is the image darkening in circular gradient from the image centre to its borders [41-42], due to light obstruction and differences in light path in some parts of the optics combination (lens + filters + neutral glasses). For the selected data set, it corresponds to a mean digital number decrease of about 5% in the visible bands and 35% in the infrared one. Higher vignetting in the infrared is mostly due to the additive high-pass filter in front of the optical device. In regards with the low radiometric variations observed from one micro plot mean to another (about 30 digital numbers), correction of this effect before extracting any quantitative data from the images seems highly important.

As vignetting has been studied for a long time in most applications of photography, from microscopy [e.g. 43] to astronomy [e.g. 44] scales, several methods have been developed to compensate for it [e.g. 45-46-47-48-49-29-50-51]. The most widely used deals with the acquisition of a flat field image, which means an acquisition over a uniform region, such as brightness variations can solely be attributed to vignetting [52-53-47]. Suitable image conditions for this approach, however, can be difficult to produce because of uneven illumination and camera tilt. Indeed, the vignetting measurements are only valid under the same camera settings, which are challenging to obtain with UAVs. Therefore, we have applied a basic three-step method usually adopted in astronomy, for instance by [54]: vignetting characterization of each sensor with an illumination radial profile, antivignetting filter production, and application of the filter to the images.

**Figure 5.**
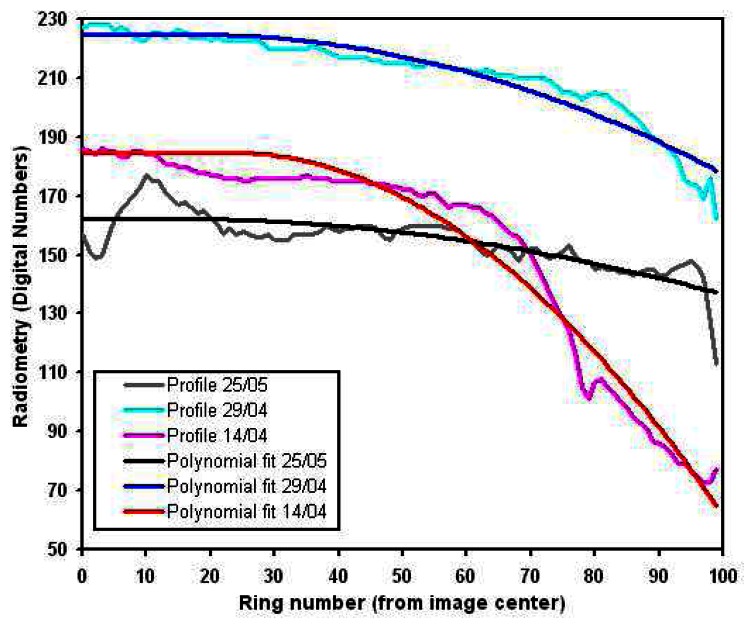
Examples of radial illumination profiles derived from the infrared images series, and their polynomial interpolations.

**Figure 6.**
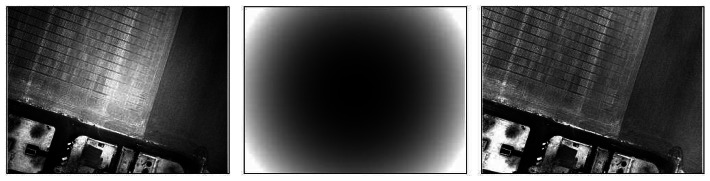
Example of result obtained by the vignetting correction process: original image (left), anti-vignetting filter (middle), and corrected image (right).

1) The radial profile is derived from the mean image of all the images acquired by a single camera in the same conditions (focal distance, aperture, shutter speed), to avoid specific objects radiometric variations contribution. This mean image is split in 100, regularly spaced, concentric rings, in which the median value is calculated. The corresponding profile (the curve of the median intensity value as a function of the distance to the image centre, cf. Figure 5) is then interpolated by a two degrees polynomial function to discard the residual noise.
2) A circular image is produced, based on concentric rings. Each ring is given the value of the illumination profile polynomial fit corresponding to its distance to the centre. The number of rings depends on the size of the image to be corrected and is equal to the half of its largest dimensions to respect the Nyquist sampling theorem. A rectangle window then truncates the circular image to fit the original image dimensions, producing the vignetting image. We have chosen to lighten the corners of the image rather than darken the centre to preserve the overall image dynamics as much as possible: the area to be modified is smaller in this case. The antivignetting filter is thus the inverse of the vignetting image, produced by subtracting it to 255, minus its minimum to fix the filter minimum to zero.
3) The antivignetting filter is applied to the image to be corrected, simply summing these two images pixel to pixel. We here assume that, before summing, highest values lay near the image centre, and lowest values at the corners. Regarding the choice of corners lightning, we chose to avoid possible saturation by interpolating pixels values between the summed image minimum and the original image maximum. This interpolation also allows preserving radiometry temporal variation. Figure 6 illustrates the result obtained with this vignetting correction method.

To prevent from color variations occurring in the visible image corners while working band by band, the visible image set is transformed from RGB to HLS system. Then we apply the same correction method than for the infrared, but only on the L band. Finally, we transform the corrected HLS image to the RGB system.

#### 3.1.2. Bidirectional reflectance effects correction

Surface reflectance varies with the incidence and view angles, following the Bidirectional Reflectance Distribution Function (BRDF) [55-56-57]. A given object will thus reflect different intensities of light in different directions. This well-known effect in aerial images is emphasized while using UAV's because they are flying at low altitudes with large field of view (compared to high resolution satellite data): the observation configurations, and especially the view angles, corresponding to each different object of a single image are then increased. As the intensity of radiometric variations due to this phenomenon is comparable to the expected variations due to crop agronomic status, we have to correct it too. Unfortunately, using our simple system impeded us to get any measurement of these configuration parameters and thus to derive the BRDF.

Therefore, we have developed a method that regularizes the received light quantity on any part of the image. We have first tested a homogenization method, named Local Range Modification [58-59], and some adaptations of this latter. Results displayed too strong radiometric changes compared to the original images to be relevant. We have thus added to this method a complex re-sampling, following five consecutive steps:

1) Sub-sampling of the original image. Blocks of pixels are averaged by means of a bilinear interpolation. This method has the advantage to be regular and reproducible, and rather independent of the strong dominant objects radiometry. Best results were obtained with scale factors equal to 200, thus sub-sampling for example a 2000× 2000 pixels image to a 10×10 pixels image.
2) Gaussian filtering on a 3×3 pixels window. To process all the pixels, even the first and last lines and rows of the image, we have first duplicated them, filtered the new image, and discarded them off the resulting image.
3) Over-sampling to the original size by bicubic interpolation. We have chosen to perform a bicubic interpolation because the bilinear interpolation does not translate the radiometric variations in the image with enough accuracy to provide an appropriate correction of the effects contained in the images. Minimum Curvature Splines, Thin Plate Splines, and Krigeage were also tested but resulted in too smooth results, and thus inaccurate corrections, along with a high consumption of processing time and complicated parameterization.
4) Inversion of the resulting image by subtracting it to 255, then scaling it to null origin.
5) Application of the resulting filter to the original image.

**Figure 7.**
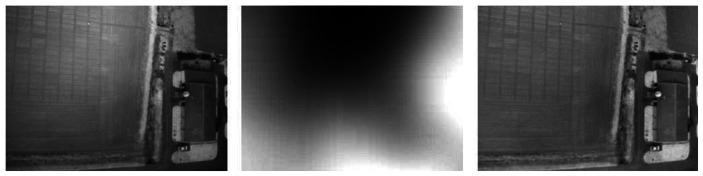
Example of result obtained by the radiometric homogenization of bidirectional effects: original image (left), homogenization filter (middle), and corrected image (right).

Although this correction method is not optimum for the whole set of images, especially for those acquired at lower altitude, it appeared to be quite good for the overall data (Figure 7).

Like for vignetting correction, band-by-band directional corrections of the visible bands result in color alterations. So, we also proceed with a RGB-HLS transformation, L-band correction only, and HLS-RGB forward transformation.

#### 3.1.3. Geometric corrections and georeferencing

UAV images, compared to satellite data for instance, have the inconvenience of lacking geographical metadata (i.e. attitude parameters) and suffering from important geometric deformations. To acquire data at a very high spatial resolution, up to 5cm/pixel, UAV have to fly at very low altitude: from 20 to 100 meters. This altitude does not provide stable conditions and the spatial resolution thus might differ from one shot to another during a single flyby. In addition, the platform instability leads to the acquisition of each image with slightly different viewing angles. We have chosen for this study to correct these different effects only by means of the georeferencing process, performed by warping any image on the basis on a single reference image for all the dates and frames [60-61-62]. This reference image was acquired on the 29/05/2005 and covered all the micro plots, with the resolution of 10cm/pixel. First, it was geographically referenced by means of fifty Differential-GPS measurements scattered over the frame. Then, about thirty ground control points were selected independently for each image to be corrected. Finally, the warping was performed independently for each image, using a second-degree polynomial fit and a bilinear resampling. Results have a root mean square error of only one to three pixels, which is acceptable compared to micro plot size (several tenths of pixel). Indeed, the buffer used to extract micro plot data will be of three pixels at least (see section 3.2).

#### 3.1.4. Intra-date and date-to-date radiometric calibration

BRDF causes radiometric variations inside a single image and thus generates differences between a given object radiometry on two consecutive images: the observation configurations are not equivalent for the two acquisitions. This effect was corrected based on common features comparison. For each date independently, we have selected for reference the most homogeneous image displaying the largest common area with the others. We have then extracted a dozen of plots, chosen to cover the largest possible radiometric scale, and derived their mean radiometric value. Bare soil was avoided because of its high sensibility to directional effects. The linear regression between the reference and the image to correct was then derived out of these values, and the resulting function applied to the whole image. Once all the images corrected, we produced one single mosaic per date per multispectral channel. The image limits for the assembling were defined following the path separating two micro plots, so that any micro plot is covered by a single image.

We performed the date-to date calibration using the same indirect calibration method, but based on invariant features like roads, parking areas, traffic paintings on roads, and buildings. We assumed the results are correct because of the very high correlation coefficients obtained for any calibration linear regression (more than 90%) and the coherent histogram variation for the whole scene over the period.

We have chosen to keep the data in digital numbers rather than transform them into reflectance data because of the challenging task that would have represented the spectral correspondence between camera channels and field spectrometric measurements. Therefore, it limits data analysis to relative methodologies, without any reference to other data, acquired either with the present or other systems. Moreover, we will derive only normalized vegetation indexes to avoid results bias due to calibration effects. Considering the scope of the present study, dedicated to show the potential of a simple design on board light UAV for crop monitoring before going on towards larger projects, we consider this limit as reasonable.

### 3.2. Quantitative data extraction

Ground-truth trial plots were accurately delimited with a differential GPS, and inserted in a vector-layer to be displayed over the mosaic-image. We have then calculated the mean radiance of each plot considering a buffer of forty centimetres, corresponding to four pixels, in order to limit the analysis area to the centre of the plot. This buffer allows discarding possible georeferencing errors and image distortions, micro plots geometric fluctuations, and avoids the inclusion of soil pixels. Averaging decreases residual errors of the various corrections (cf. section 3.1). Moreover, it is consistent with ground-truth biophysical measurements that were compiled as means for each micro plot. Vegetation indices calculated in the next section were derived from the plot mean radiance.

### 3.3. Biophysical parameters estimation

A large set of vegetation indices have been analyzed since the development of remote-sensing [63] to provide information about crop biophysical status. Commonly, indices implying infrared and red bands are related with biomass, canopy structure, and LAI, while others implying only visible bands are related with the leaf pigment concentration and nitrogen content (Table 2). An exponential relationship is usually assumed between a biophysical parameter K and the vegetation index I to which it is correlated, in the form:

(1) K=Aexp(B×I)+C

where A, B, and C are the coefficients to be derived [23-64-65-66]. These parameters have to be calibrated for the specific vegetation type and image acquisition conditions any time such relationship is to be used.

We first calculated the mean NDVI, SAVI, GNDVI, and GI (Table 2) from the mean radiance of each plot were the biophysical parameters LAI and QN were measured in the fields (cf. section 2.2). We then applied a linear interpolation between two measurements date to derive LAI and QN at each UAV acquisition date. Finally, we sought a relationship of the form (Eq.1) for each of the height couples (K; I), with K∈ {LAI, QN} and I∈ { NDVI, SAVI, GNDVI, GI }, using the “R” software. Two directions were explored:

1) A generic expression for any date and any genotype of wheat, taking into account the four acquisitions: 1/04, 14/04, 29/04, and 25/05, based on 192 ground-truth plots.
2) Date-specific expressions, reliable for each given date independently among 1/04, 14/04, 29/04, and 25/05, based on 30 to 50 ground-truth plots depending on the date.

## 4. Results and discussion

### 4.1. Generic relationships between LAI vs. NDVI, and QN vs. GNDVI

**Figure 8.**
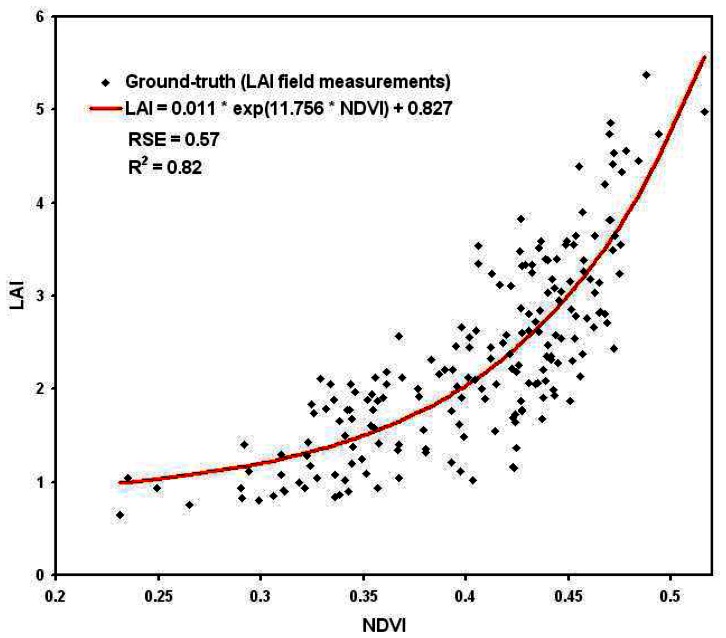
Relationship between micro-plot mean NDVI and crop mean leaf area index (LAI)

**Figure 9.**
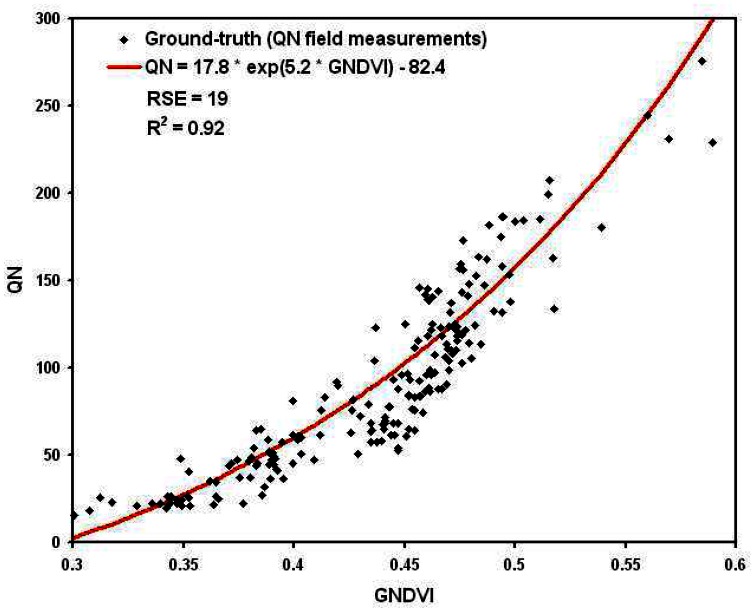
Relationship between micro-plot mean GNDVI and crop mean nitrogen uptake per square meter (QN)

The best relationships were obtained between, on one hand, the leaf area index (LAI) and the normalized difference vegetation index (NDVI) (Figure 8), and, on the other hand, the total nitrogen uptake per square metre (QN) and the green normalized difference vegetation index (GNDVI) (Figure 9):

(2) LAI=0.011exp(11.756×NDVI)+0.827

(3) QN=17.8exp(5.2×GNDVI)−82.4

Root Square Error (RSE) of the determination of these relationships were respectively 0.57 for LAI and 19 for QN, corresponding respectively to a mean relative error of 17% and 13%. This error falls down to 8% for higher LAI values (**∼**6 m^2^/m^2^) and 6% for higher QN values (**∼**300 Kg/ha). A very good correlation was found between the values calculated with these relationships and the values measured in the fields, respectively of 0.82 for LAI and 0.92 for QN.

These results are quite comparable with those usually obtained in remote sensing [e.g. 6-12-63-66-68], showing the potential of UAVs' acquisitions despite the wide range of corrections applied to the data. The obtained accuracy lies in the range of the destructive measurements precision too.

### 4.2. Date-specific relationships between LAI vs. NDVI, and QN vs. GNDVI

**Figure 10.**
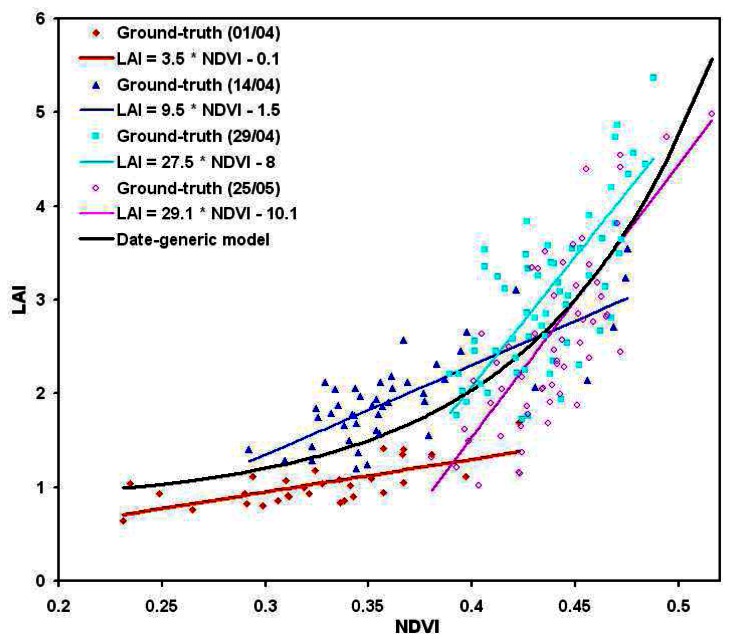
Date-specific relationships between micro-plot mean NDVI and crop mean leaf area index (LAI) for each acquisition, compared to the generic relationship.

**Figure 11.**
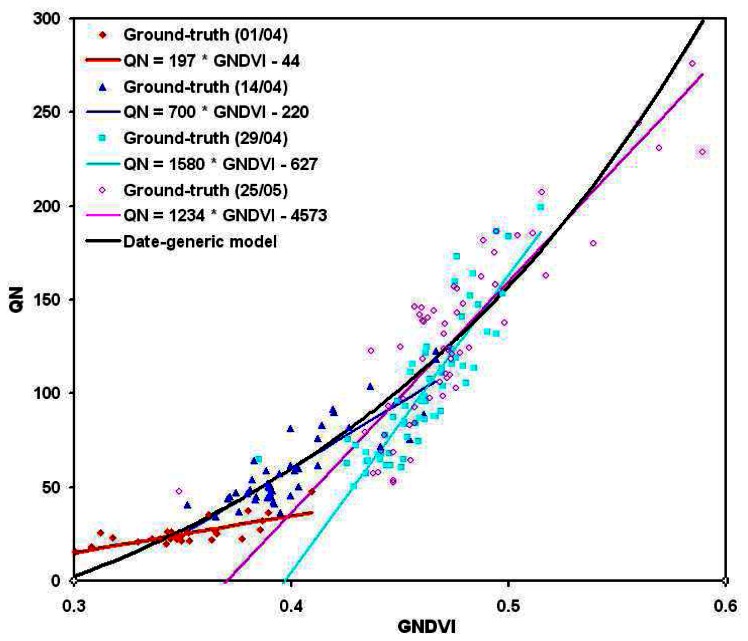
Date-specific relationships between micro-plot mean GNDVI and crop mean nitrogen uptake per square meter (QN), compared to the generic relationship.

No converging exponential model of the form (Eq.1) was found to relate either LAI to NDVI, or QN to GNDVI at a specific given date. Indeed, in the small range of values occurring for each date, these relationships are rather linear (Figure 10, Figure 11), of the form:

(4) K=A×I+B

A and B coefficients are given for each date in Table 4, together with the corresponding model errors and the coefficient of correlation between estimated and ground truth parameters. These results clearly show that either LAI or QN estimations are loosing in quantitative accuracy while using date-specific relationships. Indeed, the mean relative error in QN estimation is increasing from 13% with the generic relationship to 16-18% with the date-specific one. LAI estimation depends on the considered date: for the two middle dates, close to the flowering phenological stage, the error is a little bit lower (15-16% against 17%), while it increases up to 23% for the earlier and later dates. This effect is often observed in the literature (e.g. 22, 69, 70, 71), and gives some confidence in the validity of the observed trends.

In the global point of view, the overall values estimated date by date are slightly more correlated to the ground-truth measurements: the coefficient of correlation of the whole data set is 0.86 (against 0.82) for LAI and 0.93 (against 0.91) for QN. However, this gain is too small to justify the use of such date-specific relationships for the whole season monitoring, in regards with the loss of accuracy (higher mean relative error) the number of parameters to tune (e.g. height coefficients, compared to three).

### 4.3. Cross-validation and sensibility analysis

We have chosen to present in this paper only the evaluation of the two generic relationships, because they are the more relevant. A way to estimate the quality of the results in terms of stability and applicability is to analyze the sensibility of the derived relationship coefficients to the learning data set. In addition, comparison of calculated and ground-truth parameters on a data sample that was not used to derive the relationship is a common validation protocol. Therefore, we produced ten couples of {learning, validation} data sets, randomly splitting the whole population in two parts in the respective proportions of two thirds and one third. For each of these couples, we derived on the learning sample: “A”, “B”, “C” coefficients of (Eq. 1), and “RSE”, the mean error committed by the model. Then we applied the corresponding relationship to the related validation sample, and we calculated the coefficient of correlation between resulting values and ground-truth measurements of this validation sample “CC Valid.”. Results are given in Table 5 for LAI and Table 6 for QN estimations. These tables also give, for all the tests, the mean LAI or QN derived on the validation data set, to be compared to the ground truth data set means, respectively 2.33 m^2^/m^2^ and 88 Kg/ha. The mean difference observed between the new calculated LAI and QN of the validation sample and the generic relationship results are indicated respectively under “DLAI” and “DQN”.

Variations of the three coefficients of the exponential relationship between LAI and NDVI on one hand, and QN ad GNDVI on the other hand, are very small, showing a good stability of the model. In addition, the error of the model increase a little, due to the reduction of the sample on which it is derived. Still, it remains under in the limit of 0.6 m^2^/m^2^ for the LAI and 20 Kg/ha for QN.

Values of the two variables obtained with the tests relationships are also very close to those obtained with the generic one, difference between both being less that 0.1 m^2^/m^2^ for the LAI and less than 10 Kg/ha for QN. Moreover, the mean values derived for both LAI and QN on the total validation sample is very stable and almost equal to the means of the ground-truth measurements.

In addition, the coefficients of correlation between the derived and the measured values decrease slightly, due to the reduction of the sample like for the model residual error, but it stays very stable and high (upper than 0.79 for LAI and upper than 0.89 for QN).

**Figure 12.**
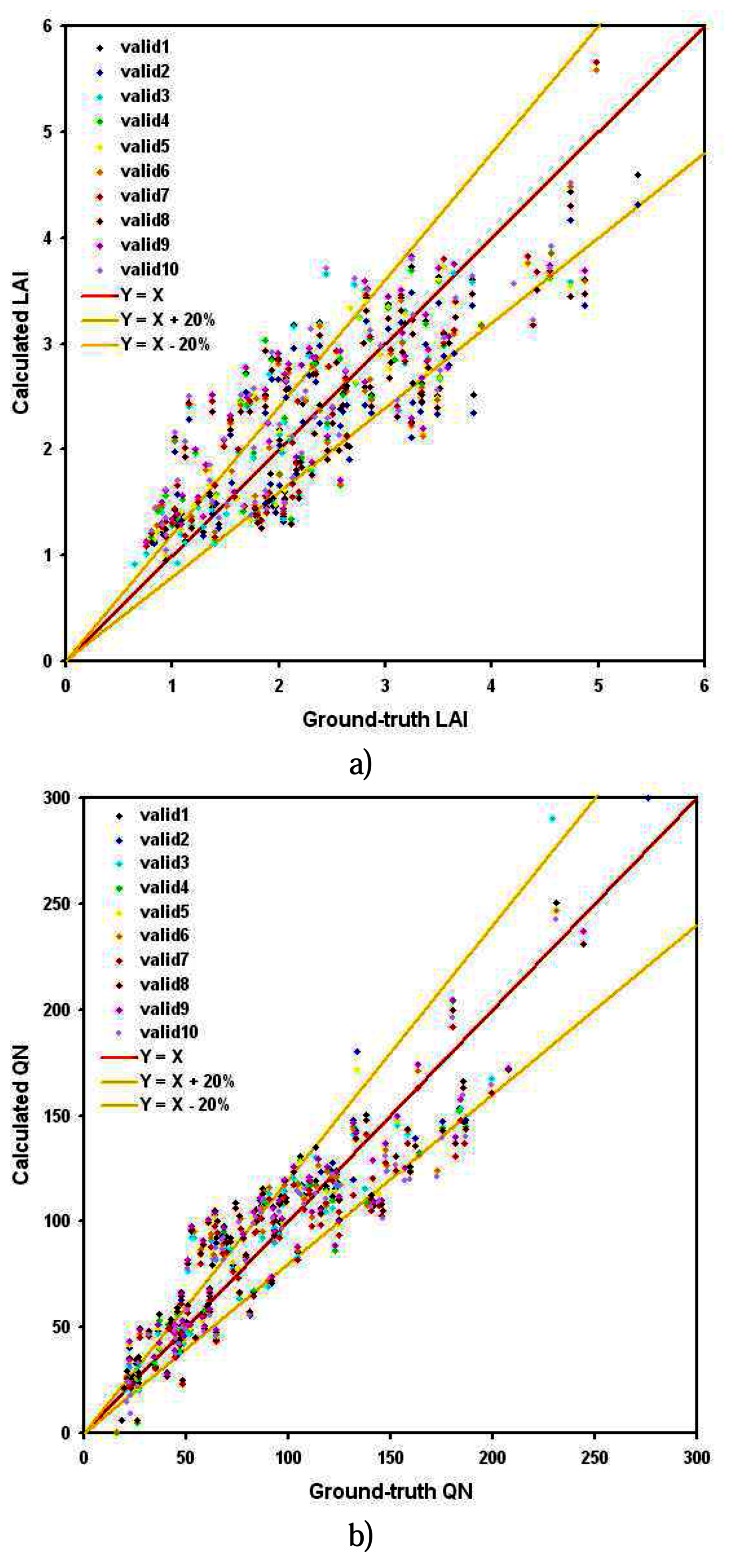
Comparison between ground truth measurements and a) LAI and b) QN, calculated by means of the different validation tests relationships.

Figure 12 shows LAI and QN distributions for the 10 validation samples, and their correlation with the ground-truth values. This distribution is quite compact and shows a good clumping around the 1:1 line. It shows that there is no artefact due to the random selection of the two sets of individuals, respectively for learning and for validation, and that each derived relationship gives results quite close to the actual values without a systematic over or under-estimation.

In conclusion, this sensibility analysis shows that the generic relationships derived at section 4.1 is very stable, does not depend on the choice of the training data set among the completely available measurements, and is quantitatively accurate.

### 4.5. LAI and QN maps

**Figure 13.**
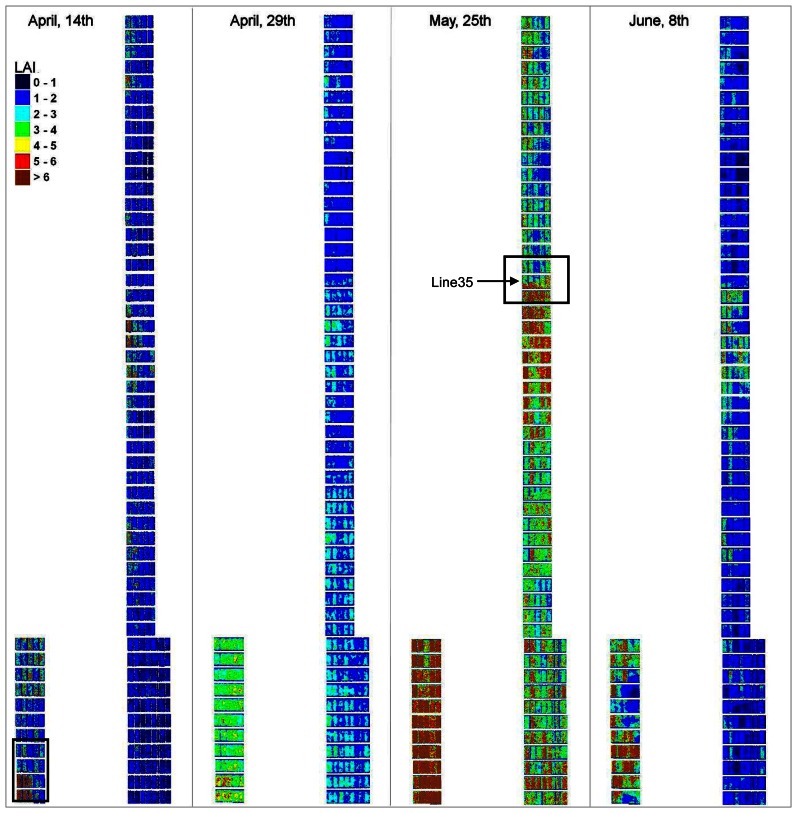
Sequence of leaf area index maps, obtained over the wheat growing-season using the derived generic relationship between NDVI and LAI (black frame indicates the zoom presented at Figure 15).

**Figure 14.**
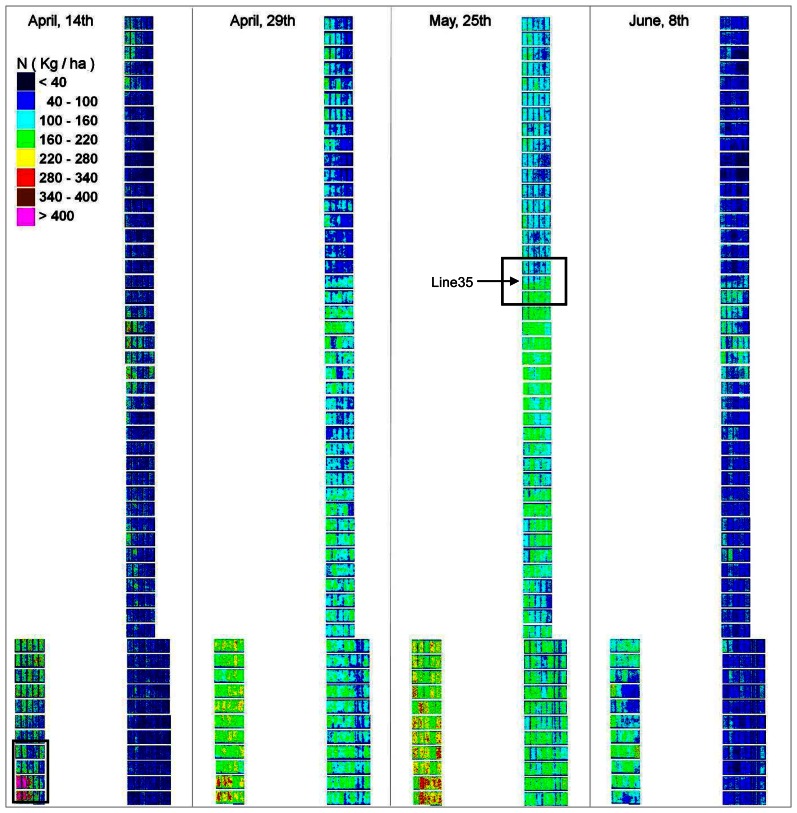
Sequence of nitrogen uptake maps, obtained over the wheat growing-season using the derived generic relationship between GNDVI and QN (black frame indicates the zoom presented at Figure 15).

We have applied the two relationships derived over the whole season to each pixel of the final mosaics of the four last dates, when LAI and QN have reached significant values. It results in time series of LAI and QN over the growing season at the intraplot scale (Figure 13 and Figure 14). The overall time-variation of these two parameters seems coherent with expected evolution through the wheat growing-season: increase until the end of May, followed by a strong decrease due to maturity and senescence. Quantitative values also seem in accordance to what is generally observed in wheat fields, except that the higher values are slightly overestimated (>6 for LAI and >400 for QN).

Other maps can be produced, deriving the biophysical parameters out of the median NDVI and the median GNDVI over the micro plot, and assigning the corresponding “median” LAI or QN to the whole plot. In some cases, this procedure could decrease the strong overestimation due to aberrant pixels.

**Figure 15.**
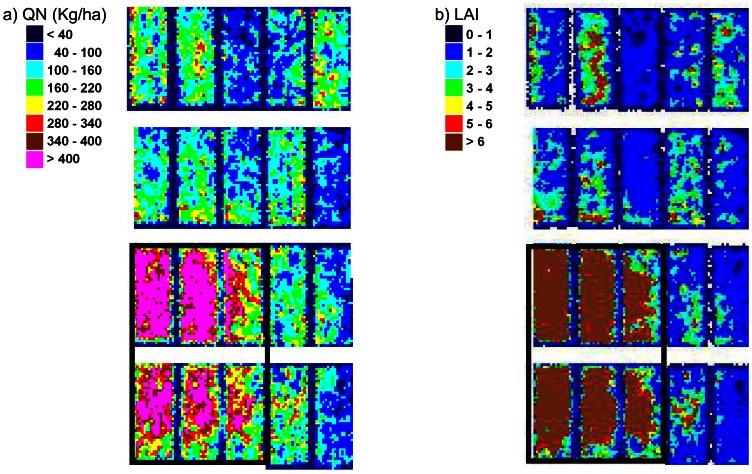
Zoom in the biophysical parameters maps on April, the 14^th^, over atypical plots at the bottom left-hand size corner of the trial showing very high QN and LAI values.

Such maps can help analyzing the different wheat varieties response to the same environment, under different fertilizing conditions. It can also provide information about the soil resources prior to the crop, especially at early stages of wheat growth. For instance, on the 14^th^ of April, a square patch of very high values appears on the bottom left-hand side corner (plot number from left to right and bottom-up: 0101, 0102, 0103, 0201, 0202, and 0203) of both LAI and QN maps (Figure 13, Figure 14, and Figure 15). This is not an artefact due to the image pre-processing, as it is explained by field factors: this part of the trial was inadvertently placed on an area that was managed as bare soil for over a year ending in October 2003. Therefore, it had a much higher level of soil nitrogen availability. This is also in good agreement with the plant sampling data in the fields (Table 7). This heterogeneity vanished as the growing season moved on and with subsequent fertilizer applications, as the surrounding area corresponded to the non-limited treatment for nitrogen.

**Figure 16.**
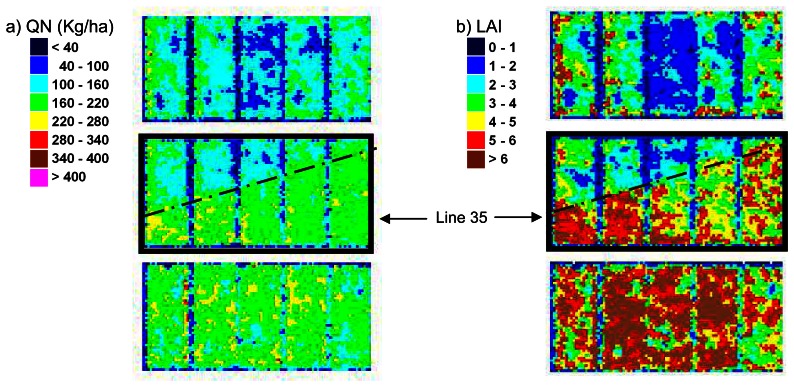
Zoom in the biophysical parameters maps on May, the 25^th^, over atypical line of plots (line 35) of the trial, showing a diagonal pattern of QN and LAI values distribution.

In addition, the fine resolution of the maps derived at the pixel scale (20cm/pixel) allows getting very accurate information to analyze the intraplot variability. For instance, a diagonal pattern appears on the five plots on row 35, for both LAI and QN, slightly on the 29^th^ of April and 8^th^ of June (Figure 13 and Figure ) and very clearly on the 25^th^ of May (Figure 16). These plots are indeed buffer plots between two nitrogen treatments. On the lower part of the trial (row 34 to 22), a nitrogen dressing (ammonium nitrate) of 50 kgN/ha as was applied mechanically on the 6^th^ of April. On the upper part (row 36 to 52) no nitrogen was applied. The image of the 14/04 was acquired too early to display the effect of nitrogen, but this latter is obvious on the following dates (29/04, 25/05 and 08/06) for LAI as well as for QN. The diagonal pattern in the buffer line is due the nitrogen spraying method used.

## 5. Conclusion and perspectives

A cost-effective multispectral sensor was designed based on commercially available digital cameras, adapted with relevant filters. It was fitted on light UAVs to perform six aerial acquisitions of wheat crop micro plots during the growing season, at very low altitude. Resulting images need several preprocessing before use, to correct vignetting, geometric and resolution-related problems, directional effects, and radiometric intercalibration. Some efficient methods were adapted for this purpose, with quite good results, and we were able to produce a coherent mosaic at the spatial resolution of 10cm/pixel, in four spectral bands: blue (420-510 nm), green (490-580 nm), red (570-650 nm), and near infrared (720-850 nm). Mean vegetation indexes were then derived for each trial micro plot and compared to ground-measured biophysical parameters. A relationship between NDVI and LAI on one hand, and GNDVI and QN on the other hand, was derived with respective RSE of 0.57 m^2^/m^2^ and 19 Kg/ha, and 82% and 92% of respective correlation between calculated and ground-truth values. These relationships were evaluated using a cross validation and a sensibility analysis to the learning data set, and proved to be quite stable and accurate. The quality of the derived relationships also shows that spectral ranges reached by standard cameras are suitable for remote sensing, and that the data pre processing is quite effective, even if it might be improved.

At the end, we were able to produce LAI and Nitrogen Uptake maps for the whole field of wheat including the trial, at any acquisition date, with an average precision of about 15% on the quantitative values, indeed in the range of the destructive measurements precision. Such maps were proved temporally and spatially coherent, even at the intraplot scale. Indeed, it shows relevant information about the intraplot variability that was explained by cropping or trial practices.

Such study thus shows that cost-effective UAV multispectral devices are relevant for quantitative wheat monitoring with a good precision. Nevertheless, many technical aspects can be improved, like the location of spectral bands (choice of near infrared filter), the characterization for the spectral sensibility and the potential for reflectance calibration. Data pre-processing are quite complex and the data quality would be improved if some of them could be avoided thanks to more stable platforms or laboratory pre-calibration of sensors. In the thematic point of view, other relationships should also be explored to evaluate the possibility of estimation of different agronomic parameters through various other indices, to increase the crops characterization. Deeper investigation of the genotype effects should also be considered in the future.

## Figures and Tables

**Figure 4.**
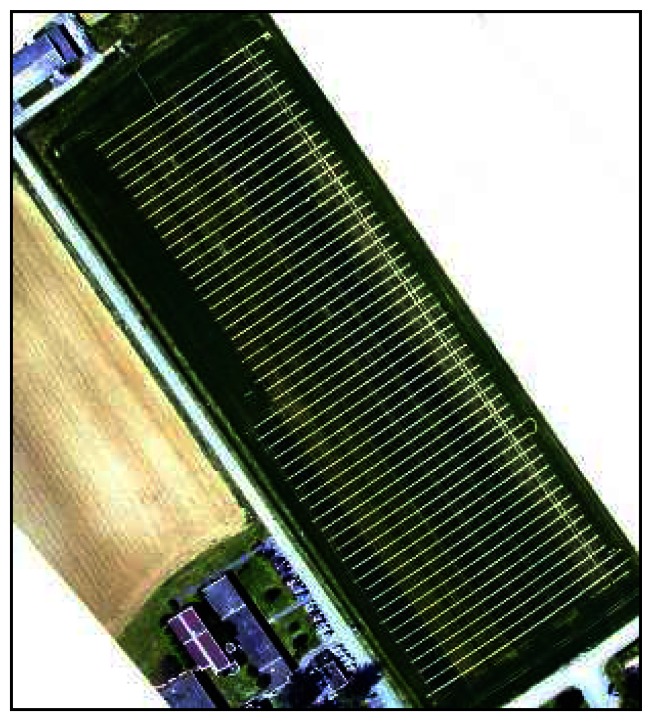
Example of UAV's acquisition (29^th^ of April, 2005) covering the whole trial field, displayed in the true Red-Green-Blue color-composition.

**Table 1.** Spectral ranges corresponding respectively to red (R), green (G), blue (B), and near infrared (NIR) channels of the two cameras used in this study, compared to common satellite sensors.

Channel	Canon EOS-350D	Sony DSC-F828	Landsat, Quickbird, Ikonos	SPOT
R	570-640 nm	570-650 nm	630-690 nm	610-680 nm
G	490-580 nm	490-570 nm	520-600 nm	500-590 nm
B	420-500 nm	430-510 nm	450-520 nm	-
NIR	720-850 nm	720-850 nm	760-900 nm	790-890 nm

**Table 2.** Mathematic expression of the vegetation indices analyzed in this study:

Name of Indices tested in this study	Index expression	Reference
NDVI (normalized difference vegetation index)	(NIR – R)/(NIR + R)	[63]
SAVI (soil-adjusted vegetation index)	(1 - L)(NIR – R)/(NIR + R - L) with L=0.5	[67]
GNDVI (green normalized difference vegetation index)	(NIR – G)/(NIR + G)	[20]
GI (greenness index)	(R – V)/(R + V)	[66]

**Table 3.** Errors of estimation and correlation coefficients between calculated and actual values for LAI and QN:

Derived parameter	RSE	Mean relative error	Min. relative error	Correlation coefficient
LAI	0.6 m^2^/m^2^	17%	8%	0.82
QN	19 Kg/ha	13%	6%	0.92

**Table 4.** Coefficients (A and B) of the date-specific linear expression relating LAI to NDVI, and QN to GNDVI, for each date. RSE is the corresponding root square error of the model, and MRE the mean relative error:

***Parameter***	***Date***	***A***	***B***	***RSE***	***MRE***
***LAI***	01/04	3.5	-0.1	0.2 m^2^/m^2^	19%
	14/04	9.5	-1.5	0.3 m^2^/m^2^	15%
	29/04	27.7	-8.0	0.5 m^2^/m^2^	16%
	25/25	29.1	-10.1	0.6 m^2^/m^2^	23%
***QN***	01/04	197	-44	4 Kg/ha	16%
	14/04	700	-220	11 Kg/ha	18%
	29/04	1580	-627	17 Kg/ha	17%
	25/25	1234	-457	23 Kg/ha	18%

**Table 5.** Results of the sensibility analysis on the estimation of LAI by means of NDVI. A, B, and C are the respective coefficients of the exponential relationship. RSE is the mean error committed on LAI with the corresponding model, in m^2^/m^2^. MLAI is the mean derived LAI for the validation data set, in m^2^/m^2^. DLAI is the mean difference between the test result on the validation sample and the generic result, in m^2^/m^2^. CC Valid. is the coefficient of correlation between the LAI calculation on the validation sample and the measured ground-truth.

LAI vs NDVI	A	B	C	RSE	MLAI	DLAI	CC Valid.

Initial	0.011	11.76	0.83	0.57	2.34	0	0.83

Test 1	0.019	10.69	0.70	0.56	2.34	0.02	0.81
Test 2	0.012	11.42	0.78	0.53	2.33	0.14	0.81
Test 3	0.014	11.32	0.73	0.57	2.32	0.02	0.79
Test 4	0.019	10.65	0.72	0.56	2.34	0.02	0.78
Test 5	0.016	10.99	0.76	0.58	2.34	0.01	0.82
Test 6	0.008	12.34	0.93	0.57	2.36	0.02	0.86
Test 7	0.01	11.97	0.85	0.59	2.35	0.06	0.85
Test 8	0.014	11.22	0.73	0.57	2.33	0.01	0.8
Test 9	0.012	11.61	0.86	0.57	2.32	0.08	0.81
Test 10	0.016	11.03	0.80	0.57	2.34	0.06	0.83

**Table 6.** Results of the sensibility analysis on the estimation of QN by means of GNDVI. A, B, and C are the respective coefficients of the exponential relationship. RSE is the mean error committed on QN with the corresponding model, in Kg/ha. MQN is the mean derived QN for the validation data set, in in Kg/ha. QN is the mean difference between the test result on the validation sample and the generic result, in Kg/ha. CC Valid. is the coefficient of correlation between the QN calculation on the validation sample and the measured ground-truth.

QN vs GDVI	A	B	C	RSE	MQN	DQN	CC Valid.

Initial	17.8	5.2	-82.4	19	89	0	0.93

Test 1	20.8	4.9	-88.1	20.5	90	1.93	0.93
Test 2	11.7	5.9	-68.1	19.3	87	3.66	0.92
Test 3	13.3	5.6	-70.4	20.4	92	4	0.92
Test 4	18.6	5.1	-86.3	20.3	91	6.7	0.92
Test 5	23.2	4.7	-96.7	19.6	91	4.07	0.89
Test 6	20.7	4.9	-89.8	19.9	88	1.07	0.9
Test 7	18.5	5	-82.2	19.9	91	9.84	0.91
Test 8	22.7	4.7	-93.9	18.7	87	4.44	0.9
Test 9	19.6	5	-85.7	19.8	90	4.49	0.92
Test 10	17.7	5.1	-80.4	19.6	88	4.4	0.92

**Table 7.** Comparison of LAI (in m^2^/m^2^) and QN (in Kg/ha) biophysical parameters between the left bottom ‘bare soil corner’ plots (grey cells) and the same cultivars on the nearest plot, on the 14^th^ of April:

Plot	Cultivar	Measured LAI (06/04)	Interpolated LAI (14/04)	Calculated mean LAI (14/04)	Measured QN (06/04)	Interpolated QN (14/04)	Calculated mean QN (14/04)
0101	25	1.7	2.7	3.5	55	143	251
0404	25	1.1	1.6	1.8	36	61	59
0102	24	1.2	2.1	3.1	44	76	219
0605	24	0.8	1.2	1.5	27	58	81
0103	23	1.2	2.1	2.5	36	72	91
0502	23	1.2	1.9	1.8	39	76	88
0201	5	2.4	3.5	3.8	78	118	124
0504	5	1.4	2.5	2.0	44	168	156
0202	4	2.3	3.2	3.7	78	123	150
0505	4	1.1	1.9	1.5	38	152	160
0203	3	1.9	3.1	2.8	55	184	166
0602	3	1.4	2.3	1.8	43	149	148
